# Standardization of the Food Composition Database Used in the Latin American Nutrition and Health Study (ELANS)

**DOI:** 10.3390/nu7095373

**Published:** 2015-09-16

**Authors:** Irina Kovalskys, Mauro Fisberg, Georgina Gómez, Attilio Rigotti, Lilia Yadira Cortés, Martha Cecilia Yépez, Rossina G. Pareja, Marianella Herrera-Cuenca, Ioná Z. Zimberg, Katherine L. Tucker, Berthold Koletzko, Michael Pratt

**Affiliations:** 1 Commitee of Nutrition and Wellbeing, International Life Science Institute (ILSI-Argentina), Buenos Aires C1059ABF, Argentina; 2 Facultad de Ciencias Medicas, Departamento Nutricion, Universidad Favaloro, Solís 453, Buenos Aires C1078AAI, Argentina; 3 Fundação Jose Luiz Egydio Setubal, Hospital Infantil Sabara, Instituto Pensi, São Paulo 01239-040, Brazil; 4 Centro de Atendimento e Apoio ao Adolescente, Departamento de Pediatria, Universidade Federal de São Paulo, São Paulo 04023-062, Brazil; 5 Departamento de Bioquímica, Escuela de Medicina, Universidad de Costa Rica, San José 11501, Costa Rica; 6 Centro de Nutrición Molecular y Enfermedades Crónicas, Departamento de Nutrición, Diabetes y Metabolismo, Escuela de Medicina, Pontificia Universidad Católica, Santiago 833-0024, Chile; 7 Departamento de Nutrición y Bioquímica, Pontificia Universidad Javeriana, Bogotá, Colombia; 8 Colegio de Ciencias de la Salud, Universidad San Francisco de Quito, Quito 17-1200-841, Ecuador; 9 Instituto de Investigación Nutricional, La Molina, Lima 12, Peru; 10 Centro de Estudios del Desarrollo, Universidad Central de Venezuela (CENDES-UCV)/Fundación Bengoa, Caracas 1010, Venezuela; 11 Departamento de Psicobiologia, Universidade Federal de São Paulo, São Paulo 04023-062; 12 Department of Clinical Laboratory and Nutritional Sciences, University of Massachusetts Lowell, Lowell, MA 01854, USA; 13 University of Munich Medical Center, Division of Metabolic and Nutritional Medicine, Dr. von Hauner Children’s Hospital, Ludwig-Maximilians-Universität München, D-80337 Munich, Germany; 14 Nutrition and Health Sciences Program, Hubert Department of Global Health, Rollins School of Public Health, Emory University, Atlanta, GA 30322, USA

**Keywords:** nutrition, Latin America, food composition database, standardization

## Abstract

Between-country comparisons of estimated dietary intake are particularly prone to error when different food composition tables are used. The objective of this study was to describe our procedures and rationale for the selection and adaptation of available food composition to a single database to enable cross-country nutritional intake comparisons. Latin American Study of Nutrition and Health (ELANS) is a multicenter cross-sectional study of representative samples from eight Latin American countries. A standard study protocol was designed to investigate dietary intake of 9000 participants enrolled. Two 24-h recalls using the Multiple Pass Method were applied among the individuals of all countries. Data from 24-h dietary recalls were entered into the Nutrition Data System for Research (NDS-R) program after a harmonization process between countries to include local foods and appropriately adapt the NDS-R database. A food matching standardized procedure involving nutritional equivalency of local food reported by the study participants with foods available in the NDS-R database was strictly conducted by each country. Standardization of food and nutrient assessments has the potential to minimize systematic and random errors in nutrient intake estimations in the ELANS project. This study is expected to result in a unique dataset for Latin America, enabling cross-country comparisons of energy, macro- and micro-nutrient intake within this region.

## 1. Introduction

The global epidemic of obesity in all age groups and in both developed and developing countries has raised the need for large-scale multicenter studies on dietary/lifestyle factors. These studies are essential to substantially improve our knowledge on the complex relationship existing between energy imbalance, obesity and associated chronic diseases at large scale and wider geographical heterogeneity [1].

Evaluating dietary consumption is an arduous task. Food intake is a complex phenomenon and its assessment using available tools is subject to errors inherent to the instruments themselves, as well as the interviewee and the interviewer. These errors, defined as systematic and random, should be known and controlled in order to generate more precise and powerful data to reveal risks associated to unhealthy dietary patterns [2].

Increasingly, literature has been published on the methodological and statistical approaches to improve the standardization of dietary measurements collected in large multicenter studies [1,3,4,5]. A major operational issue relates to the comparability of dietary measurements collected from populations of different countries. Between-country comparisons are particularly prone to error when different food composition databases and software are used to estimate nutrient intake [4,5]. Food composition tables are rarely consistent across countries and many foods are defined or presented in different ways, making comparisons of the dietary intake difficult.

Although there have been ongoing efforts since 1984 to standardize food composition databases over the world [6], the lack of a homogeneous and complete Latin American database represents a handicap for nutritional epidemiology studies on the relationship between nutrition and health, and the comparison and evaluation of dietary intake within this world region, similar to the situation for international studies in other parts of the world.

The process of standardization at the food and nutrient assessments was performed to minimize systematic and random errors in nutrient intake estimations and to allow comparisons between Latin American countries involved in ELANS (Latin American Nutrition and Health Study). Therefore, the aim of this paper is to describe the procedures and rationale for the selection and adaptation of food composition from a single database during the ELANS study to make cross-country nutritional intake comparisons.

## 2. Experimental Section

### 2.1. Study Sample

The Latin American Study of Nutrition and Health/*Estudio Latinoamericano de Nutrición y Salud* (ELANS) is a household-based multi-national cross-sectional survey aimed at investigating food and nutrient intake as well as nutritional and physical activity statuses of nationally representative samples from urban populations. The project involves eight Latin American countries (*i.e.*, Argentina, Brazil, Chile, Colombia, Costa Rica, Ecuador, Perú, and Venezuela) representing a total cohort of 9000 individuals, aged 15.0–65.0 years, stratified by geographical location (only urban areas), gender, age, and socioeconomic status. The rationale and design of the study are reported in more detail elsewhere [7]. The overarching ELANS protocol was approved by the Western Institutional Review Board (#20140605) and is registered at Clinical Trials (#NCT02226627). Each site-specific protocol was also approved by the ethical review boards of the participating institutions. 

### 2.2. Dietary Assessment

Dietary intake data were obtained using two 24-h food recalls (24-HR) performed on two non-consecutive days within one week, totaling a target of 18,000 24-HR recalls. 24-HR was selected because of its nearly universal applicability across populations with varying literacy skills and its relatively low burden for participants. As a single 24-HR is limited and generally inadequate for assessing the diet of individuals, two recalls were chosen to estimate routine food consumption and to evaluate intra-individual variability in nutrient intake [8].

24-HRs provided detailed information on all food and beverages (including water and alcoholic beverages), preparations and supplements consumed over previous 24 h, or usually, the previous day [9]. Information collected included the time of consumption, the name of the eating occasion, detailed food descriptions allowing accurate food coding, and the amount eaten. Separate forms were included to report on homemade recipes so name of dish, total quantities of all ingredients and fraction of dish consumed could be stated.

Each recall was administered in person by trained interviewers, provided with standardized neutral probing questions according to the Multiple Pass Method [10] to improve the precision of the information obtained. To assist the participant in specifying and quantifying foods in household measures, a photographic album containing the most commonly household utensils and size portions were used. These were specific to each country, including local food item pictures and common utensils, and standardized within the country.

The household measures obtained in the 24-HR were converted to grams (g) and milliliters (mL) by trained nutritionists, according to the literature and/or previously standardized references in each country. Then, this information was transformed into energy, macronutrient, and micronutrient quantities using the Nutrition Data System for Research software, version 2013 (NDS-R, Minnesota University, MN, USA). NDS-R is an accurate nutrient and food group serving calculation software available for research purposes, which is based on the United States Department of Agriculture (USDA) Nutrient Data Laboratory as the primary source of nutrient values and nutrient composition, including over 18,000 foods. Among the 150 nutrients available in NDS-R, 19 were initially prioritized in the ELANS based on their importance in the diet and on fact that they had the most complete information in the database (Table 1).

**Table 1:** Energy, micro and macronutrient final outcomes in Latin American Study of Nutrition and Health (ELANS).

Nutrients (Unit)	
Energy (kcal)	
Macronutrients	Total protein (g)
	Total carbohydrate (g)
	Total fat (g)
Micronutrients	Vitamin A (RAE)
	Vitamin D (μg)
	Vitamin C (mg)
	Calcium (mg)
	Iron (mg)
	Sodium (mg)
Fiber (g)	
Added sugar (g)	
Animal and vegetable proteins (g)	
Saturated fatty acids (g)	
Monounsaturated fatty acids (g)	
Polyunsaturated fatty acids (g)	
Trans-fatty acids (g)	
Cholesterol (mg)	

### 2.3. Quality Control in Coding of Reported Foods/Beverages

A quality control system to minimize error and increase reliability of interviewing and coding 24-HR was performed in all study-sites, according to a theoretical framework presented elsewhere [3]. All the interviewers were trained using a standard form for the application of the 24-HR and an explanatory manual for completing this form. All recalls were checked by a dietitian within three to eight days after the interview. If there was any problem with data quality (e.g., handwriting, out-of-range quantities, recipe not described), the interviewee was contacted in order to provide the information and/or to verify the information in the second recall, and when not resolved, the recall was excluded.

Researchers of each country analyzed the consistency of the data according to the review of the quantities of key nutrients (kilocalories, carbohydrates, protein, and fat). High or low intakes were rechecked and verified, or the recall was designated inaccurate. If no reasonable explanation could be ascertained, the recall was designated as unsatisfactory and the participant was excluded.

### 2.4. Food Matching

**Figure 1:**
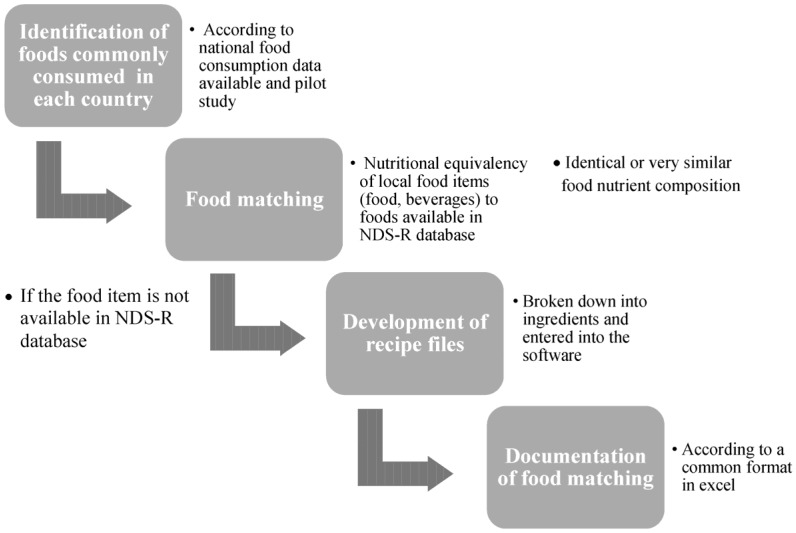
Standardized food matching procedure applied across Latin American Study of Nutrition and Health (ELANS) countries.

NDS-R was chosen for ELANS to increase comparability of nutrient composition of foods consumed by study participants. As NDS-R was generated in the USA, a food matching standardized procedure was strictly conducted by professional nutritionists in each country in both 24-HR (Figure 1). This harmonization process included a check of nutritional equivalency of food items reported by the study subjects of each country against the USA version of foods available in NDS-R database; if the food item was not available in the NDS-R database, a food with similar definition, description, and nutrient content was considered. To ensure maximum similarity with the Latin American foods, USA foods were identified in the NDS-R database through its general description (*i.e.*, by name, type and mode of preparation). The values described in the software were compared to the values available in local food composition tables (Table 2) or nutrition labels and food industry composition tables. A concordance rate between 80 and 120% for energy and macronutrient content was required to accept a food selection from this database [11]. Some examples of food matching performed in ELANS are presented in Table 3.

**Table 2:** Local food composition tables used in the food matching process.

Country	Local Food Composition Tables *
Argentina	Latin American Network of Food Composition Data System (LATINFOODS) [12]
Brazil	Tabela Brasileira de Composição de Alimentos [13]
Chile	Tabla de Composición Química de los Alimentos Chilenos [14]
Colombia	Tabla de Composición de Alimentos Colombianos [15,16]
Costa Rica	Tabla de Composicin de Alimentos de Centroamerica [17], Infonut [18]
Ecuador	Tabla de Composición de Alimentos Colombianos [15], Tablas de Uso Práctico de los Alimentos de Mayor Consumo en México [19], Tablas Peruanas de Composición de Alimentos [20], Llanos *et al.* [21]
Peru	Tabla de composición de alimentos del Instituto de Investigación Nutricional [22] (includes the Tablas Peruanas de Composición de Alimentos [20]),
Venezuela	Tabla de Composición de Alimentos para Uso Práctico [23]

* All countries also utilized food labels from manufacturers when foods were not available in any database.

**Table 3:** Examples of food matching and recipes created in NDS-R for ELANS.

Food/Recipe	Ingredients
**Food**	
Aji colorado molido (peper)	Peppers, hot chili, red, raw
Anón (fruit)	Sugar apple (sweetsop or anon)
Arroz doce (sweet rice)	Desserts, miscellaneous, pudding, rice (arroz con leche), plain
Avena a granel (oat bulk)	Ingredient, cooked cereal—dry, oat bran, before cooking
Caldo de frijol (beans soup)	Soup, bean, black beans, prepared from ready-to serve can, regular
Feijão-carioca (beans)	Beans, brown, canned—drained, regular, boiled, salt—regular, fat used as seasoning—oil, soybean-unhydrogenated
Goiabada (sweet)	Candy, other confections, dulce de guayaba (sweetened guava paste)
Kafta (meat)	Meatball, without sauce, beef, hamburguer or ground beef—10% fat (90% lean meat)
Mote cocido (corn)	Corn, white, cooked from fresh, whole kernel
Ponqué con sabor a ron (cake)	Hispanic, ponque (rum flavored pound no frosting)
Queque de tienda (cake)	Cake, yellow or flavored, purchased ready-to-eat, not frosted or glazed, after cooking
Semillas de chan (seeds)	Seeds, chia
Tacaco (Sechium tacaco)	Potato, raw, with refuse
**Recipe**	
Açaí (fruit)	Fresh figs; soybean oil
Bolo de laranja (cake)	Egg, raw, whole; sugar, white granulated; juice or flavored drink, orange, juice, fresh; oil, soybean—unhydrogenated; baking powder, regular; and flour, white all-purpose, enriched
Buriti (fruit)	Egg, raw, yolk only; oil, soybean—unhydrogenated; orange, fresh
Chanfainita (mixed dish)	Potato, boiled, without skin, before cooking; peppers, hot chili, red, raw; vegetables, garlic, fresh; onion, white, yellow or red, raw; beef, organ meats, kidney; oil, soybean 90%/cottonseed 10%; mint, spearmint, fresh
Chicha (beverage)	Flour, corn, masa, yellow—enriched; brown sugar; cloves (ground); cinnamon (ground)
Cholao (beverage)	Banana, fresh or ripe; apple, fresh, with skin; papaya, fresh; passion fruit (maracuya)—fresh; pineapple, fresh; nuts and seeds coconut, fresh; strawberries, fresh; kiwi green; condensed (sweetened), regular; jams or preserves, regular; juice or flavored drink, lemonade and lemon drinks, homemade
Coucus (mixed dish)	Water, tap; cornstarch; cornmeal, dry, yellow (degermed, enriched)
Empanadas de pipian (pastries)	Flour, corn, masa, white—enriched; oil, soybean—unknown type, tomato, cooked from fresh; onion, white, yellow or red, cooked, eggs boiled; cornstarch; potato, boiled, without skin; nuts and seeds peanuts, roasted, dry roasted, unsalted; peppers, sweet red, raw
Ensalada de verduras cocida sin mayonesa (salad)	Carrots, raw; vegetables, corn, white, cooked from fresh, cob; broccoli, raw, string or green beans; raw, lemon, juice, fresh
Gallo pinto (rice and beans dish)	Rice white, regular cooking, cooked in salted water; onion, white, yellow or red, cooked, after cooking edible portion; peppers sweet red cooked, beans black, cooked form dried, after cooking salt
Picadillo de papa (potato dish)	Potato boiled with out skin; peppers sweet red cooked; onion with, yellow or red edible portion after cooking; cilantro fresh, salt
Plátano maduro con queso (plantain with cheese)	Plantains, ripe yellow, boiled or baked after cooking, no salt added; cheese queso fresco (Mexican white cheese, margarine regular stick salted, soybean palm oils)
Pudim de coco com calda de caramelo (pudding)	Eggs, boiled; coconut, dried (shredded or flaked), unsweetened; coconut, milk, fresh (liquid from grated meat—water added); milk, condensed (sweetened), regular; milk, whole (3.5%—4% fat); sugar, white granulated
Sopa de fideos con verduras (soup)	Spaguetti noodles, white, cooked in unsalted water; potato, boiled, without skin, before cooking; carrots, raw; celery, raw; leeks, raw; squash, hubbard, before cooking

No new food was added to the NDS-R database and no chemical analyses were performed. Regional foods, recipes, and commercial foods not available in the NDS-R database were broken down into ingredients and entered into the software as user recipes. These user recipes were created from the available NDS-R database and documented in a food-matching control sheet. They were provided by national publications, recipe books, and culinary websites of each country, and checked against actual data from 24-HR.

When regional foods did not have an exact equivalent or similar food available in the NDS-R database, one or more foods combined were inserted as a recipe, as the software does not accept “new foods”. Local teams were responsible for creating a recipe that represents the same nutritional value as the original version. Some examples of recipes are described in Table 3. The list of recipes is documented as part of the ELANS procedure of food matching.

Before beginning the field study, each country study group standardized approximately 600 food/beverages and recipes commonly consumed by its population, according to ELANS pilot study and other available sources of national food consumption data.

### 2.5. Data Consistency

After the end of the fieldwork and prior conducting the analysis of food intake, data consistency was held for each 24-HR to ensure that there was no error of typing or food entered and to ensure more reliable results. Initially it was performed the consistency of energy intake. Total daily intake values below 800 kcal/day or above 4000 kcal/day suggested errors in collection or data entry and were revised. Then, the total intake of micronutrients was analyzed. The micronutrient composition of the detailed participant´s food intake with extreme values was compared with the same national food composition tables used for the verification of energy and macronutrient content. If the concordance rate was not between 80% and 120%, a routine was performed in Stata version 13 (StataCorp, College Station, TX, USA) for correction of the values.

## 3. Conclusions

Until now, no study has evaluated the food and nutrient intake, as well as nutritional status and physical activity patterns of representative populations in Latin America using a standardized methodology across a consortium of several participating countries. The Latin American Study of Nutrition and Health/*Estudio Latinoamericano de Nutrición y Salud* (ELANS) is a randomized, cross-sectional, multicenter investigation on nutrition and physical activity of adolescents and adults in eight Latin American countries.

The standardization of nutritional assessment in ELANS should prevent or minimize bias (systematic errors) which could affect the pooling of data collected in different centers and improve between-country comparisons.

Full information on the general and specific criteria applied in this harmonization of food composition database in ELANS will be freely available and will provide comparability with similar studies to be performed in other countries.
